# Family Functioning and Adolescent Delinquency in Mainland China: Positive Youth Development Attributes as a Mediator

**DOI:** 10.3389/fpsyt.2022.883439

**Published:** 2022-04-28

**Authors:** Daniel T. L. Shek, Kim H. Leung, Diya Dou, Xiaoqin Zhu

**Affiliations:** Department of Applied Social Sciences, The Hong Kong Polytechnic University, Kowloon, Hong Kong SAR, China

**Keywords:** family functioning, positive youth development, delinquency, Chinese adolescents, mediator

## Abstract

According to the positive youth development (PYD) approach, PYD attributes such as psychosocial competencies are developmental assets which can promote the holistic development of adolescents, such as increase in thriving and decrease in risk behavior. Although there are research findings supporting this theoretical proposition, there are several weaknesses in this literature. These include a lack of studies examining family antecedents of PYD attributes and the mediating role of PYD attributes in the relationship between family functioning and adolescent delinquency. There are also few longitudinal studies utilizing large samples and validated measures of family functioning and PYD attributes in the scientific literature. In this study, we examined the predictive effect of family functioning on adolescent delinquency and the mediating role of PYD attributes. Using a short-term longitudinal study in Sichuan, China, we collected two waves of data from 4,981 adolescents aged 11 and above, with 6 months between the two waves. Analyses using structural equation modeling showed that family functioning at Wave 1 negatively predicted the level of and change in delinquent behavior at Wave 2, with PYD attributes at Wave 2 as a mediating factor. The present study enriches the conceptual framework on the role of family functioning and PYD attributes in adolescent delinquent behavior. The findings also suggest that strengthening family functioning and PYD attributes would protect adolescents from engaging in delinquent acts.

## Introduction

Research findings show that adolescent delinquency is a growing concern in different parts of the world. Johnston et al. (1) stated that vaping marijuana doubled or tripled in 8th, 10th, and 12th United States graders across 2017 to 2019 based on the “Monitoring the Future” survey. Besides, vaping nicotine showed a sharp increase over the same interval, rising from 11.0% in 2017 to 25.5% in 2019 among 12th United States graders. Ministry of Justice (2) reported that the reoffending rate of United Kingdom children and adolescents in 2018 (38.5%) was higher than young adults (27.5%) or adults (28.0%) based on the results of Youth Justice Statistics. Hirschmann (3) stated that there was an increase of almost 4% of the 3-year recidivism rate of juvenile offenders from 2014 to 2019 in Singapore.

Based on the ecological perspective (4) and general systems approach (5), researchers have proposed that individual factors and environmental factors (such as family factors) contribute to juvenile delinquency. Different social scientists have also used the strain theory, social control theory and social learning theory to explain adolescent delinquency (6). For example, in Reckless’s (7) containment theory, both personal and social environmental attributes are proposed as antecedents of adolescent delinquency. In particular, the role of the family as an environmental factor in influencing adolescent delinquency has been well-documented in the Western literature [e.g., (8, 9)]. In this study, we made reference to Reckless’s containment theory (7) and Lerner and Castellino’s developmental contextual model (5) to investigate how the family (interpersonal factor) and positive attributes of adolescents (intrapersonal factor) contribute to adolescent delinquency.

With reference to China, although there are studies showing the effects of personal attributes, such as self-control (10) and self-efficacy (11), as well as social environmental factors such as bonding with parents (12) and family conflict (13) on adolescent delinquency, they are mainly cross-sectional studies and the inter-relationships amongst family factors (such as family functioning), adolescent developmental assets [such as positive youth development (PYD)] and adolescent delinquency are not systematically examined (14). As such, the present study investigated how family functioning and PYD attributes predict delinquent acts of Chinese adolescents over time, with PYD attributes proposed as a mediator.

### Family Functioning and Adolescent Delinquency

The family is commonly viewed as an important determinant of adolescent delinquency [e.g., (15, 16)]. In general, family functioning refers to the overall health of the family environment and quality of interactions among family members (17). Different family functioning models generally uphold the thesis that healthy family functioning is the basis for healthy adolescent development (18–20). With particular reference to China, family is also regarded as an important foundation shaping adolescent developmental outcomes (21–23).

Past research has revealed that family functioning was negatively associated with adolescent delinquency regardless of gender and country. Yun and Cui (24) revealed that parental warmth was negatively associated with delinquency over time in both American (*N* = 5,665) and Korean (*N* = 3,438) adolescents in their cross-cultural study, although the protective effect of parental warmth was stronger for American adolescents than Korean counterparts. Besides, Sánchez-Queija et al. (25) revealed that family cohesion and parental attachment predicted less substance use over time in 513 adolescents in Spain regardless of gender. Estrada et al. (26) utilized a randomized controlled trial method to assess the impact of a parent-centered preventive intervention on delinquent acts of 746 Hispanic adolescents. Results showed the preventive effect of family intervention on adolescent drug use and unsafe sexual activities. Unfortunately, few studies have examined the relationship between family functioning and adolescent delinquency based on Chinese adolescents (27–29).

### Positive Youth Development and Adolescent Delinquency

In contrast to the deficit view of adolescence which mainly focuses on pathologies and problems of adolescents, the PYD approach is concerned with promotion of positive development, enhancement of plasticity, development of competencies and facilitation of personal growth of adolescents (30). Theoretically, different PYD approaches highlight the importance of PYD attributes (e.g., 40 developmental assets in Peter Benson’s theory, 5Cs in Richard Lerner’s model, and 15 PYD attributes identified by Richard Catalano) in promoting adolescent developmental outcomes (30). In this study, we conceived PYD attributes based on the work of Richard Catalano who identified 15 PYD attributes from effective PYD programs in the United States (31). These included bonding (development of strong affective relationship with people and institutions), social competence (development of interpersonal skills), emotional competence (awareness of one’s own emotions and capacity for emotion regulation), cognitive competence (cognitive abilities, processes, or outcomes), behavioral competence (ability to use strategies to perform socially acceptable behavior and to make effective behavior choices), moral competence (ability to make ethical judgment and to perform ethical behaviors), self-efficacy (beliefs in one’s abilities to perform certain acts and achieve goals), prosocial norms (establishment of clear and healthy guidelines and standards for prosocial behaviors), resilience (ability to adapt changes in a healthy way), self-determination (ability to set goals and make choices based on own thinking), spirituality (development of the belief in a higher power and cultivation of a sense of life meaning and values), beliefs in the future (development of hope and optimism), clear and positive identity (building of self-esteem and a sense of self), prosocial involvement (participation in prosocial behaviors), and recognition for positive behavior (getting rewards and recognition of positive behaviors). Based on the Chinese Positive Youth Development Scale (CPYDS; 32), 15 positive youth attributes are subsumed under four higher-order factors, namely, cognitive-behavioral competencies, prosocial attributes, positive identity, and general PYD qualities. These attributes are viewed as important assets to promote favorable cognitive and psychosocial developmental outcomes of adolescents.

The general prediction of the PYD approach is that PYD attributes would be negatively related to adolescent delinquency (14). Previous cross-sectional studies have provided empirical evidence on the negative associations between PYD constructs and adolescent delinquency. Pechorro et al. (33) examined the moderating role of self-control in the aggression-delinquency link of 567 Portuguese adolescents using structural equation modeling (SEM) techniques. They revealed the negative associations between self-control and delinquency and conduct disorder outcomes of adolescents. They also found the moderating effect of self-control on the negative impact of different forms of aggressive acts on delinquency and conduct disorder outcomes. Apart from the moderating role of PYD, Wang et al. (34) found the negative contribution of regulatory emotional self-efficacy as a PYD construct to school bullying and the significant indirect effect of regulatory emotional self-efficacy on the link between self-esteem and school bullying of 995 adolescents in Xi’an, China.

Moreover, McDaniel (35) utilized a pretest-posttest quasi-experimental research design to assess the effectiveness of self-determination training in short-term detention facilities over 6 months. The training aimed to foster the sense of self-determination and motivation, and teach goal-setting to the youth. The results revealed that the rate of recidivism reduced compared to the average rate of youth detained in the short-term facilities. In the meta-analytic study conducted by Beelmann and Lösel (36), the interventions targeted to promote social skills have been found to associate with reduction of aggression, delinquency, and related antisocial outcomes of children and adolescents in randomized controlled trials. There are also limited studies showing the value of PYD attributes in reducing juvenile delinquency. In the Project P.A.T.H.S. in Hong Kong, longitudinal data collected over 5 years revealed that those joining the P.A.T.H.S. program showed slower growth in delinquency and intention to engage in delinquent behavior as compared to the control participants (37).

### Mediators Between Family Functioning and Adolescent Delinquency

Some studies have examined the mechanisms underlying the relationship between family functioning and adolescent delinquency. Consistent with the spillover hypothesis, Liu et al. (13) revealed that marital discord and interparental inconsistency contributed to impaired adolescent mental health and attachment with their parents, as well as an increase in delinquent peer association in 2,496 Chinese adolescents, which in turn increased their likelihood of engaging in delinquent acts. In other words, poor family functioning results in delinquent behaviors of adolescents via poor parental attachment and engagement in delinquent peer groups. Similarly, Du and Kim (38) found that poor family functioning contributed to parental depression, which subsequently resulted in the increase in delinquent behaviors of 450 American adolescents.

Besides, Mwangangi (15) proposed that the family serves as an important foundation for character, value, and skill development of the child, which in turn determines his/her delinquent acts at later times. To our best knowledge, very few studies have been conducted to examine the effects of positive family functioning processes on adolescent delinquent acts via characters and values. Jin et al. (39) revealed that the positive effect of interparental intimacy on interpersonal adjustment of both 554 delinquent and 344 non-delinquent Chinese adolescents was partially mediated by filial piety. In addition, Walters (40) assessed the mediating effect of self-efficacy on the paths from parental support and monitoring to delinquency in 2,252 adolescents in Columbia. The findings revealed that self-efficacy for a conventional lifestyle served as a mediator of the parenting-delinquency relationship, where parental control and support help shaping a child’s self-efficacy, which further reduces the likelihood of the child’s engagement in future delinquent acts.

Several longitudinal and intervention studies have also been conducted to examine the mediating role of PYD attributes in the family functioning-delinquency link. Watts (41) investigated the mediating role of social bond in the path from child abuse and neglect at Wave 1 to delinquency at Wave 2 in 9,002 American adolescents. He found that social bond at Wave 1 negatively contributed to later delinquency, and the deteriorating effect of child abuse and neglect on delinquency was completely mediated by social bonds among females. Similarly, Walters (42) conducted a longitudinal path analysis to assess the mediating role of moral engagement and competencies at Wave 1 in the path from involvement in structured community activities at Wave 0 to violent acts at Wave 2 of 1,170 male juvenile offenders. The findings not only indicated the negative association between moral engagement and violent acts, but also revealed the negative indirect effect of involvement in structured community activities on violent acts via moral engagement. As research on the mediating role of PYD in the link between family functioning and adolescent delinquency is still sparse and limited to one or few PYD constructs [e.g., (40, 42, 43)], the present study attempted to advance our understanding of the link between family functioning and adolescent delinquency with reference to the mediating role of PYD attributes.

There are several weaknesses of the literature surrounding the predictive effect of family functioning and PYD attributes on delinquency in Chinese adolescents. Conceptually, although there are theoretical propositions on the role of parenting (i.e., dyadic family process) in adolescent delinquency [e.g., (24)], we do not know whether family functioning (i.e., systemic family process) influences adolescent delinquency. If family functioning influences adolescent delinquency, we also do not know whether family functioning affect delinquency through other factors such as PYD? Methodologically, studies on the impact of family functioning on adolescent delinquency have been predominately conducted in Western countries, thus illustrating the need to conduct studies in Asian contexts (such as the Chinese context) to assess the generalizability of research findings. Second, as studies are predominantly cross-sectional studies, there is the need for more longitudinal studies. Third, the sample size in some studies is not large [e.g., (22, 34)], thus creating generalizability problem for the research findings. Fourth, most studies employed OLS regression to analyze data, thus suggesting the need to use more advanced statistical analyses like SEM techniques which take measurement errors of the scales into account. Fifth, although there are discrete measures of PYD attributes (e.g., self-efficacy), there are few validated measures covering a wide range of PYD attributes in the field. The development of validated measures on PTD attributes undoubtedly assist in testing the relationships amongst family functioning, PYD attributes, and adolescent delinquency. With reference to these weaknesses, we asked several research questions in this study:

Research Question 1: Is family functioning related to adolescent delinquency? Based on previous studies (24, 25), we expected that there would be a negative relationship between family functioning and adolescent delinquency (Hypothesis 1).

Research Question 2: Is family functioning related to PYD attributes? With reference to the existing findings (44, 45), it was hypothesized that there would be a positive relationship between these two domains (Hypothesis 2).

Research Question 3: Are PYD attributes related to adolescent delinquency? Based on the developmental assets theories [e.g., Catalano’s theory of 15 PYD constructs in Shek et al. (30)] and past studies (33, 34), it was expected that there would be a negative relationship between these two areas (Hypothesis 3).

Research Question 4: Do PYD attributes mediate the impact of family functioning on adolescent delinquency? Based on previous findings that PYD attributes such as bonding mediated the impact of family intactness on adolescent delinquency (46), we expected that PYD attributes would mediate the impact of family functioning on adolescent delinquency (Hypothesis 4).

## Materials and Methods

### Participants and Procedures

A longitudinal research design with data collected at two time points was utilized in this study. To understand the research questions in adolescents, we focused on the responses of adolescents aged 11 and above. The participants were 5,690 adolescents recruited from five randomly selected primary and secondary schools in Sichuan, China in 2019 (Wave 1) before the outbreak of COVID-19. Among them, 4,981 students participated in the study again after 6 months (Wave 2) after resumption of school. The attrition rate was 12.5%. On both occasions, students responded to the same questionnaire including measures of perceived family functioning, PYD, and delinquency in their classrooms during school hours. Approval for research ethics from Sichuan University and consent to participate in the study was obtained from schools, parents, and students. Key principles upheld in data collection and usage (e.g., study purpose, voluntary participation, and confidentiality) were clearly explained to the students before collecting the data. All procedures met the basic ethical standards in research involving human subjects such as harmless to subjects and respect for persons. Further details of the study can be seen in Dou et al. (47). After data collection, students’ responses at Wave 1 and Wave 2 were matched according to their names, classes, and class numbers. The final matched sample consisted of 4,922 3rd to 9th graders (51.5% females and 48.5% males; mean age was 13.1 ± 1.32 years old) at Wave 1. Most of them were Hans (99.3%), 61% of students lived in cities and 39% of students lived in rural areas.

### Instruments

The survey involved several validated measures on the psychosocial adjustment of adolescents. Among the measures, family functioning, PYD and adolescent delinquency were the focus of this study.

#### Family Functioning

The Chinese Family Assessment Instrument (C-FAI) was used to assess family functioning (48). It is composed of 33 items reflecting five domains including mutuality (12 items, e.g., family members get along well), communication (9 items, e.g., parents know children’s needs), conflict and harmony (6 items, e.g., poor marital relationship of parents), parental concern (3 items, e.g., parents love their children), and parental control (3 items, e.g., parents scold and beat children). Students were asked to answer on a 5-point scale ranging from 1 (most similar) to 5 (most dissimilar). Reverse scoring was performed on all positive items so that a higher score reflects better family functioning. Past research with Chinese students has shown that C-FAI possesses good psychometric properties (49, 50). It has high factorial validity, good reliability and invariance properties. Five dimensions of C-FAI reflect the major features of family functioning in Chinese families, including absence of conflict, mutuality, connectedness, positive parent-child relationships, and positive spousal relationship.

#### Positive Youth Development

The CPYDS was utilized to measure the positive attributes of students (51). It is composed of 80 items reflecting fifteen dimensions including bonding (6 items, e.g., When I need help, I trust my parents will help me), resilience (6 items, e.g., When I face difficulty, I will not give up easily), social competence (7 items, e.g., I can actively talk to a stranger), emotional competence (6 items, e.g., I am a pleasant person), cognitive competence (6 items, e.g., I believe there is a solution to any problem), behavioral competence (5 items, e.g., I can express views that are different from others), moral competence (6 items, e.g., I have high moral expectation about my behavior), self-determination (5 items, e.g., I am able to make wise choices), self-efficacy (2 items, e.g., I can finish almost everything that I am determined to do), spirituality (7 items, e.g., To me, life is very dull versus very exciting), beliefs in the future (3 items, e.g., I have confidence to solve my future problems), clear and positive identity (7 items, e.g., I am a filial person), prosocial involvement (5 items, e.g., In this school, students are encouraged to have voluntary service), prosocial norms (5 items, e.g., I care about unfortunate people in the society), and recognition for positive behavior (4 items, e.g., When I complete my tasks, teachers will praise me). Except the items of spirituality that were responded to a 7-point scale (1: most negative to 7: most positive), all items were answered on a 6-point scale ranging from 1 (strongly disagree) to 6 (strongly agree). A higher score indicates a higher level of PYD in this study. Previous research with Chinese students has shown that CPYDS is valid and reliable (14, 52).

#### Delinquency

A 12-item scale was used to assess the occurrence of delinquent behaviors in the students in the previous 12 months. In this study, a delinquent behavior was defined as the act that harms others and disobeys rules, regulations, or norms (53). These behaviors include fighting in gangs, stealing things, truancy, attacking other people physically, cheating at examinations, running away from home, bullying or harassing other people, damaging the properties of other people, having sex with others, speaking foul language, trespassing, and not returning home without parental permission. Students were asked to respond to a 7-point scale (0 = never, 1 = one to two times; 2 = three to four times; 3 = five to six times; 4 = seven to eight times; 5 = nine to ten times; and 6 = more than ten times). The scale has been used in previous research with Chinese students and possesses good psychometric properties (14, 54).

### Data Analysis

**FIGURE 1:**
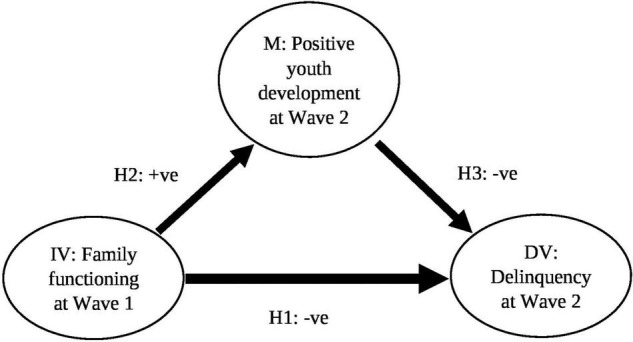
Conceptual model for the mediating effect of positive youth development at Wave 2 on the path from family functioning at Wave 1 to delinquency at Wave 2. Note: IV, independent variable; M, mediator; DV, dependent variable; +ve, hypothesized positive relationship; and −ve, hypothesized negative relationship.

As the missing rate of this study was very small (2 participants with missing data only), no imputation was performed and the analyses were based on listwise deletion. In line with the analytic approach to mediation adopted by Baron and Kenny (55), three essential conditions were tested first before developing and testing the mediation model in this study. The conditions involve the effects from family functioning at Wave 1 (independent variable) on delinquency at Wave 2 (dependent variable), from family functioning at Wave 1 to PYD at Wave 2 (mediator), and from PYD at Wave 2 to delinquency at Wave 2 when regressing delinquency at Wave 2 on both family functioning at Wave 1 and PYD at Wave 2. As the effects were significant, the mediation model aimed to assess the mediating effect of PYD at Wave 2 on the path from family functioning at Wave 1 to delinquency at Wave 2 was subsequently examined (Figure 1).

As recommended by Weston and Gore (56), the measurement portion of the mediation model was assessed before examining the structural relationships among latent variables. In this study, the measurement model was specified as (1) each latent variable (family functioning, PYD, and delinquency) was predicted by its corresponding indicators, (2) uncorrelated uniqueness was specified among the indicators, and (3) all latent variables were allowed to correlate freely. After validating the measurement model which revealed that the indicators were significantly loaded only on their corresponding latent variables, the structural portion of the mediation model was examined. All specifications in the structural mediation model were the same as those of the measurement model, except for the hypothesized predictions among the latent variables.

All effects were examined via SEM in LISREL 8.54 and estimated using maximum likelihood estimation. The model fit was assessed based on the standardized root-mean-square residual (SRMR): <0.08 = a good fit (57), the root-mean-square error of approximation (RMSEA): <0.05 = a close fit, 0.05 −0.08 = a fair fit, 0.08 −0.10 = a mediocre fit, and >0.10 = a poor fit (58), non-normed fit index (NNFI): >0.90 = acceptable fit (59), as well as comparative fit index (CFI): >0.90 = acceptable fit (59). These fit indices were selected based on the criteria stated in Brown (60). Sobel test was used to assess the significance of indirect effects. It was reasonably utilized because large sample size in this study would yield lower standard errors to the estimates (61). If 95% confidence intervals (CI) for the estimates of indirect effects did not include zero, the estimates were significant at 0.05 level (62).

To control the confounding effects of extraneous variables, age and gender were incorporated into the structural mediation model as covariates and the resulting model was tested again. Specifically, the impact of covariates on PYD and delinquency at Wave 2 were specified and estimated (63). Age and gender were chosen to control for because they have been found to associate with PYD and delinquency in previous related studies (14, 53). Besides, as the time-varying construct at Time 1 is commonly treated as the exogenous covariate to those at later occasions (63), the effect of the delinquency score at Wave 1 was also controlled for. It was achieved by incorporating the delinquent score at Wave 1 into the previous model with age and gender and the resulting model was tested again. In this study, significant effects of age, gender and delinquency at Wave 1 on delinquency at Wave 2 (age: β = 0.01, *p* < 0.01; gender: β = 0.03, *p* < 0.05; delinquency at Wave 1: β = 0.44, *p* < 0.001) and PYD at Wave 2 (age: β = −0.09, *p* < 0.001; gender: β = 0.08, *p* < 0.001; delinquency at Wave 1: β = −0.43, *p* < 0.001) were found using regression analyses. Consequently, the influence of age, gender and delinquency at Wave 1 on the mediating effect of PYD in the family functioning – delinquency link was controlled for.

## Results

### Attrition Analyses

Considering sample attrition over time, the present findings revealed that student dropout at Wave 2 was not very high (12.5%). Moreover, the matched sample showed similar socio-demographic characteristics to dropouts including age and gender. Furthermore, Little’s MCAR Test showed that the missing value of the dataset were completely at random [Little’s MCAR test: χ^2^_(296)_ = 100.84, *p* = 1.00], suggesting that attrition bias was unlikely to occur (64).

### Descriptive Statistics, Reliability, and Inter-Correlation

Table 1 illustrates the means, standard deviations, Cronbach’s alphas and inter-correlations of all study variables. Specifically, all measures were reliable (Cronbach’s alphas ranged from 0.67 to 0.98) [see (65, 66)]. As expected, all factors of family functioning at Wave 1 and PYD at Wave 2 were negatively related to delinquency at Wave 1 and Wave 2. Besides, family functioning at Wave 1 and PYD at Wave 2 were positively associated with each other.

**TABLE 1:** Descriptive statistics, reliability, and inter-correlation of study variables (*N* = 4,922).

	1	2	3	4	5	6	7	8	9	10	11	12	13	14	15	16	17	18	19	20	21	22	23	24	25	26
1. Age	–																									
2. Gender *^a^*	0.00	–																								
3. W1 DB	**0.14**	**−0.12**	–																							
4. W1 MU	**−0.13**	0.00	**−0.28**	–																						
5. W1 COM	**−0.15**	−0.03	**−0.28**	**0.83**	–																					
6. W1 CF	**−0.10**	**0.04**	**−0.21**	**0.50**	**0.44**	–																				
7. W1 PCC	**−0.06**	**0.08**	**−0.20**	**0.65**	**0.58**	**0.52**	–																			
8. W1 PCT	**−0.05**	**0.08**	**−0.19**	**0.29**	**0.31**	**0.54**	**0.39**	–																		
9. W1 FF	**−0.12**	**0.05**	**−0.30**	**0.83**	**0.80**	**0.77**	**0.80**	**0.69**	–																	
10. W2 BO	**−0.11**	0.00	**−0.21**	**0.29**	**0.32**	**0.24**	**0.22**	**0.20**	**0.33**	–																
11. W2 RES	**−0.14**	−0.02	**−0.23**	**0.28**	**0.30**	**0.26**	**0.21**	**0.18**	**0.31**	**0.76**	–															
12. W2 SC	**−0.14**	−0.01	**−0.19**	**0.26**	**0.28**	**0.23**	**0.18**	**0.17**	**0.29**	**0.63**	**0.69**	–														
13. W2 RPB	**−0.11**	0.01	**−0.20**	**0.25**	**0.27**	**0.23**	**0.19**	**0.20**	**0.30**	**0.69**	**0.66**	**0.71**	–													
14. W2 EC	**−0.09**	−*0.03*	**−0.20**	**0.27**	**0.31**	**0.23**	**0.18**	**0.19**	**0.30**	**0.61**	**0.68**	**0.74**	**0.70**	–												
15. W2 CC	**−0.18**	−*0.03*	**−0.23**	**0.29**	**0.31**	**0.25**	**0.20**	**0.19**	**0.32**	**0.63**	**0.74**	**0.76**	**0.72**	**0.78**	–											
16. W2 BC	**−0.14**	−0.01	**−0.21**	**0.26**	**0.28**	**0.22**	**0.19**	**0.17**	**0.29**	**0.61**	**0.68**	**0.71**	**0.68**	**0.70**	**0.78**	–										
17. W2 MC	**−0.13**	0.03	**−0.24**	**0.25**	**0.26**	**0.23**	**0.17**	**0.16**	**0.27**	**0.59**	**0.64**	**0.69**	**0.64**	**0.68**	**0.73**	**0.75**	–									
18. W2 SDE	**−0.16**	−0.01	**−0.21**	**0.25**	**0.28**	**0.24**	**0.19**	**0.17**	**0.29**	**0.59**	**0.69**	**0.68**	**0.62**	**0.68**	**0.76**	**0.77**	**0.74**	–								
19. W2 SSE	**−0.13**	−0.02	**−0.17**	**0.22**	**0.25**	**0.19**	**0.16**	**0.16**	**0.26**	**0.51**	**0.58**	**0.57**	**0.54**	**0.56**	**0.64**	**0.63**	**0.59**	**0.66**	–							
20. W2 CPI	**−0.18**	**−0.07**	**−0.22**	**0.28**	**0.33**	**0.23**	**0.19**	**0.18**	**0.31**	**0.60**	**0.66**	**0.68**	**0.63**	**0.69**	**0.73**	**0.67**	**0.67**	**0.71**	**0.63**	–						
21. W2 BF	**−0.20**	−0.02	**−0.23**	**0.28**	**0.31**	**0.25**	**0.20**	**0.19**	**0.32**	**0.55**	**0.66**	**0.64**	**0.58**	**0.63**	**0.71**	**0.65**	**0.64**	**0.70**	**0.59**	**0.80**	–					
22. W2 PI	**−0.19**	*0.03*	**−0.24**	**0.29**	**0.30**	**0.24**	**0.19**	**0.20**	**0.31**	**0.61**	**0.63**	**0.64**	**0.67**	**0.63**	**0.70**	**0.65**	**0.67**	**0.64**	**0.57**	**0.70**	**0.70**	–				
23. W2 PN	**−0.11**	**0.08**	**−0.22**	**0.23**	**0.23**	**0.21**	**0.19**	**0.17**	**0.26**	**0.54**	**0.58**	**0.60**	**0.61**	**0.55**	**0.64**	**0.63**	**0.63**	**0.59**	**0.51**	**0.59**	**0.61**	**0.73**	–			
24. W2 SP	**−0.13**	**−0.10**	**−0.20**	**0.32**	**0.36**	**0.27**	**0.23**	**0.25**	**0.37**	**0.51**	**0.56**	**0.49**	**0.49**	**0.59**	**0.54**	**0.46**	**0.45**	**0.47**	**0.44**	**0.62**	**0.58**	**0.52**	**0.38**	–		
25. W2 PYD	**−0.18**	−0.02	**−0.26**	**0.33**	**0.37**	**0.29**	**0.24**	**0.23**	**0.38**	**0.77**	**0.83**	**0.83**	**0.81**	**0.84**	**0.89**	**0.84**	**0.82**	**0.84**	**0.74**	**0.86**	**0.83**	**0.83**	**0.75**	**0.69**	–	
26. W2 DB	**0.10**	**−0.08**	**0.46**	**−0.19**	**−0.21**	**−0.21**	**−0.15**	**−0.20**	**−0.25**	**−0.26**	**−0.27**	**−0.26**	**−0.25**	**−0.27**	**−0.28**	**−0.26**	**−0.29**	**−0.26**	**−0.22**	**−0.28**	**−0.29**	**−0.29**	**−0.26**	**−0.31**	**−0.34**	–
Mean	13.1	−	0.30	4.18	4.04	3.97	4.37	3.84	4.08	5.13	5.18	4.84	4.98	4.67	4.99	5.08	4.88	5.06	4.87	4.64	4.89	4.94	5.22	5.50	4.99	0.28
SD	1.32	−	0.48	0.87	0.96	0.84	0.91	1.14	0.73	0.96	0.95	0.96	1.02	1.13	0.99	0.90	0.92	0.93	1.11	1.03	1.13	1.07	0.87	1.40	0.83	0.46
α	–	–	0.81	0.93	0.91	0.69	0.69	0.80	0.95	0.89	0.90	0.88	0.85	0.88	0.91	0.84	0.82	0.86	0.67	0.88	0.84	0.88	0.82	0.93	0.98	0.80
Mean inter-item correlation	–	–	0.41	0.53	0.53	0.28	0.45	0.57	0.36	0.58	0.61	0.53	0.58	0.56	0.64	0.51	0.44	0.55	0.51	0.51	0.63	0.61	0.49	0.65	0.42	0.40

*Note: Correlation coefficients in bold and in italic are significant at 0.01 and 0.05 levels, respectively. DB, delinquency; W1, Wave 1; MU, mutuality; COM, communication; CF, conflict and harmony; PCC, parental concern; PCT, parental control; FF, family functioning; BO, bonding; RES, resilience; SC, social competence; RPB, recognition for positive behavior; EC, emotional competence; CC, cognitive competence; BC, behavioral competence; MC, moral competence; SDE, self-determination; SSE, self-efficacy; CPI, clear and positive identity; BF, beliefs in the future; PI, prosocial involvement; PN, prosocial norms; SP, spirituality; PYD, positive youth development; W2, Wave 2. ^a^ 1, male and 2, female.*

### Scale Validation

In this study, Confirmatory factor analysis (CFA) was conducted to validate the first- and higher-order structures of C-FAI and CPYDS, and first-order structure of the scale for delinquency.

#### Chinese Family Assessment Instrument

The present results supported the five-factor correlated model of C-FAI with four error covariances (SBχ^2^ = 27,268.08, *df* = 481, *p* < 0.001; NNFI = 0.94, CFI = 0.95, RMSEA = 0.099, SRMR = 0.099). Factor loadings ranged from 0.29 to 0.87 and were significant at 0.05 levels. The subscales of C-FAI were reliable [composite reliability (CR) ranged from 0.72 to 0.93 and nearly all average variance extracted (AVE) were above 0.50] and the mean inter-factor correlation was moderate (*r* = 0.57). Hierarchical CFA results further supported the second-order structure of C-FAI at Wave 1 (SBχ^2^ = 28,965.74, *df* = 487, *p* < 0.001; NNFI = 0.94, CFI = 0.94, RMSEA = 0.101, SRMR = 0.106). The loadings of the second-order factor (family functioning) on five primary factors ranged from 0.41 to 0.99 and were significant at 0.05 levels. The Cronbach’s alpha of family functioning was 0.95.

#### Chinese Positive Youth Development Scale

The fifteen-factor correlated model of CPYDS was empirically supported (χ^2^ = 28,863.73, *df* = 2,975, *p* < 0.001; NNFI = 0.99, CFI = 0.99, RMSEA = 0.047, SRMR = 0.036) in this study. Factor loadings ranged from 0.47 to 0.89 and were significant at 0.05 levels. The subscales were reliable (CR ranged from 0.68 to 0.93 and nearly all AVE were above 0.50) and the mean inter-factor correlation was quite high (*r* = 0.74). Moreover, higher-order CFA results further confirmed the second-order structure of CPYDS at Wave 2 (χ^2^ = 35,715.34, *df* = 3,065, *p* < 0.001; NNFI = 0.99, CFI = 0.99, RMSEA = 0.053, SRMR = 0.045). The loadings of the second-order factor (PYD) on 15 primary factors ranged from 0.64 to 0.92 and were significant at 0.05 levels. The Cronbach’s alpha of PYD was 0.98.

#### Scale for Delinquency

The CFA results supported the unidimensional structure of the scale for delinquency at Wave 1 (SBχ^2^ = 575.27, *df* = 54, *p* < 0.001; NNFI = 0.94, CFI = 0.95, RMSEA = 0.041, SRMR = 0.068) and at Wave 2 (SBχ^2^ = 428.63, *df* = 54, *p* < 0.001; NNFI = 0.93, CFI = 0.94, RMSEA = 0.037, SRMR = 0.065). Factor loadings ranged from 0.32 to 0.79 and from 0.35 to 0.72 at Wave 1 and Wave 2, respectively. The CR of the scale was 0.90 and 0.89 at Wave 1 and Wave 2, respectively. A higher score indicates a higher level of delinquency in this study.

### Prediction of Family Functioning and Positive Youth Development on Delinquency

The present findings revealed that family functioning at Wave 1 positively contributed to PYD at Wave 2 (β = 0.36, *p* < 0.001; fit indices: χ^2^ = 63,482.4, *df* = 6,191, *p* < 0.001, NNFI = 0.98, CFI = 0.98, RMSEA = 0.052, SRMR = 0.054), but negatively contributed to delinquency at Wave 2 (β = −0.17, *p* < 0.001; fit indices: χ^2^ = 27,897.9, *df* = 936, *p* < 0.001, NNFI = 0.94, CFI = 0.94, RMSEA = 0.097, SRMR = 0.086). PYD at Wave 2 negatively contributed to delinquency at Wave 2 when regressing delinquency on both family functioning and PYD (β = −0.21, *p* < 0.001; fit indices: χ^2^ = 70,642.4, *df* = 7,599, *p* < 0.001, NNFI = 0.98, CFI = 0.98, RMSEA = 0.050, SRMR = 0.056). As such, three essential conditions for the development of the mediation model highlighted by Baron and Kenny (55) were fulfilled. Subsequently, the mediation of PYD at Wave 2 on the path from family functioning at Wave 1 to delinquency at Wave 2 were created and tested.

### Mediating Effect of Positive Youth Development on the Path From Family Functioning to Delinquency

In this study, the CFA results revealed a good fit of the measurement model to the data (χ^2^ = 70,642.4, *df* = 7,599, *p* < 0.001, NNFI = 0.98, CFI = 0.98, RMSEA = 0.050, SRMR = 0.056). This implies that two second-order latent variables (family functioning and PYD) were best represented by their corresponding first-order factors, which in turn were inferred by corresponding indicators. Similarly, delinquency was best represented by its corresponding indicators. Delinquency at Wave 2 was negatively correlated with family functioning at Wave 1 (*r* = −0.17, *p* < 0.05) and PYD at Wave 2 (*r* = −0.24, *p* < 0.05). Family functioning at Wave 1 was positively related to PYD at Wave 2 (*r* = 0.36, *p* < 0.05).

**FIGURE 2:**
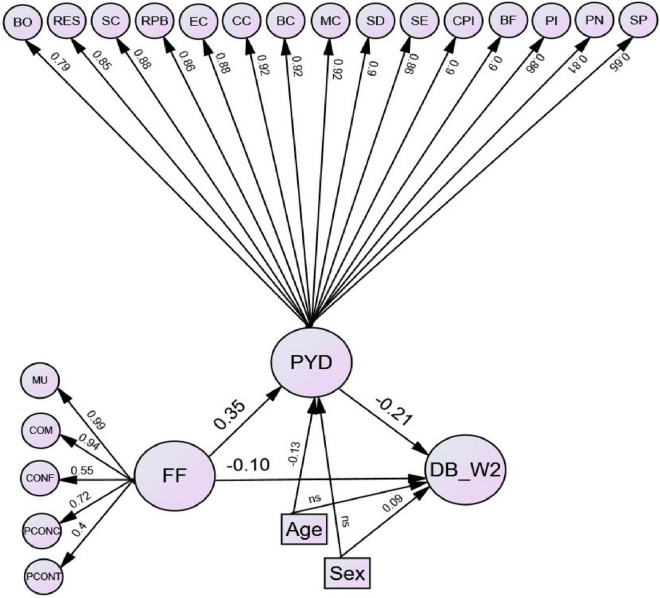
Mediation model of positive youth development at Wave 2 on the path from family functioning at Wave 1 to delinquency at Wave 2 when controlling for age and gender. Note: Standardized estimates are shown in the figure. All paths were significant at 0.05 levels except for the paths from age to delinquency at Wave 2, and from sex to positive youth development at Wave 2. Indicators of all latent variables, correlations among the factors of family functioning, and correlations among covariates are omitted for clarity. DB_W2, delinquency at Wave 2; MU, mutuality; COM, communication; CONF, conflict and harmony; PCONC, parental concern; PCONT, parental control; FF, family functioning; BO, bonding; RES, resilience; SC, social competence; RPB, recognition for positive behavior; EC, emotional competence; CC, cognitive competence; BC, behavioral competence; MC, moral competence; SD, self-determination; SE, self-efficacy; CPI, clear and positive identity; BF, beliefs in the future; PI, prosocial involvement; PN, prosocial norms; SP, spirituality; PYD, positive youth development; Age, age; Sex, sex.

Figure 2 illustrates the results of the structural mediation model controlling for age and gender. The CFA results revealed a good fit of the mediation model to the data (χ^2^ = 72,378.9, *df* = 7,843, *p* < 0.001, NNFI = 0.98, CFI = 0.98, RMSEA = 0.049, SRMR = 0.056). Family functioning at Wave 1 positively contributed to PYD at Wave 2 (β = 0.35, *p* < 0.05), but negatively contributed to delinquency at Wave 2 (β = −0.10, *p* < 0.05). PYD at Wave 2 negatively predicted delinquency at Wave 2 (β = −0.21, *p* < 0.05). The findings supported Hypotheses 1, 2, and 3.

Table 2 indicates the decomposition of effects in the structural mediation model. The indirect effect of family functioning at Wave 1 on delinquency at Wave 2 via PYD at Wave 2 was negative and statistically significant (β = −0.072, 95% CI = −0.076 to −0.068). The path coefficient of the direct effect of family functioning at Wave 1 on delinquency at Wave 2 decreased when controlling for PYD at Wave 2 (β = −0.097, *p* < 0.05) compared to the direct effect without controlling for PYD (β = −0.169, *p* < 0.05), indicating that the effect of family functioning at Wave 1 on delinquency at Wave 2 is *partially mediated* by PYD at Wave 2 [see (66)]. The indirect effect explained 42.6% of the total effect. Overall, the mediation model explained 7.4% of the variance in delinquency at Wave 2, which was higher than the proportion of variance in delinquency at Wave 2 that was explained by the direct effect model without including PYD (4.0%). The findings supported Hypothesis 4.

**TABLE 2:** Results of mediation analyses with covariates (*N* = 4,922).

Pathways	Standardized effects	95% CI (Lower bound)	95% CI (Upper bound)	Percentage of indirect effect in the total effect (%)	*R*^2^ (%)
**With covariates (age and sex)**					
***Direct effects***					
Family functioning → positive youth development	0.347	0.335	0.359	42.6	7.4
Positive youth development → delinquency	−0.207	−0.240	−0.174		
Family functioning → delinquency	−0.097	−0.109	−0.085		
***Indirect effect***					
Family functioning → positive youth development → delinquency	−0.072	−0.076	−0.068		
***Total effect***					
Family functioning → delinquency	−0.169	−0.181	−0.157		
**With covariates (age, sex, and delinquency score at Wave 1)**					
***Direct effects***					
Family functioning → positive youth development	0.321	0.292	0.350	64.0	16.7
Positive youth development → delinquency	−0.172	−0.182	−0.162		
Family functioning → delinquency	−0.031	−0.041	−0.021		
***Indirect effect***					
Family functioning → positive youth development → delinquency	−0.055	−0.059	−0.051		
***Total effect***					
Family functioning → delinquency	−0.086	−0.096	−0.076		

*Note: The effect is significant at the 0.05 level when 95% confidence interval (CI) does not contain 0.*

**FIGURE 3:**
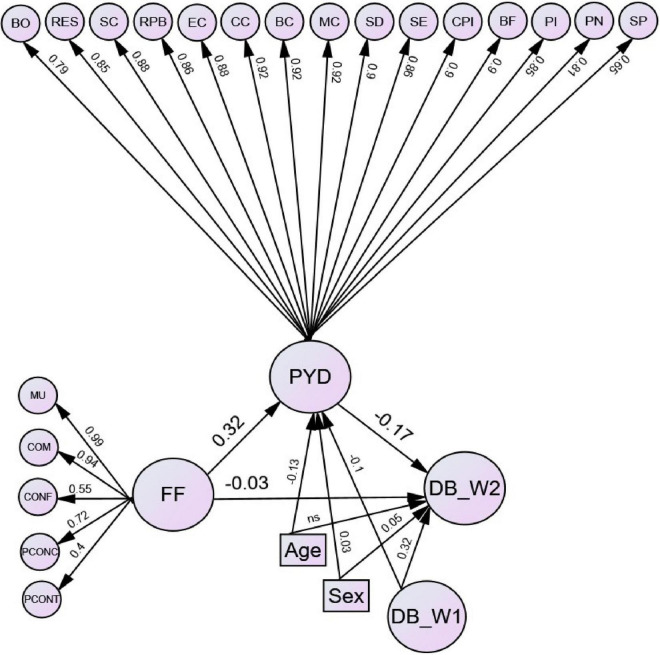
Mediation model of positive youth development at Wave 2 on the path from family functioning at Wave 1 to delinquency at Wave 2 when controlling for age, gender, and delinquency score at Wave 1. Note: Standardized estimates are shown in the figure. All paths were significant at 0.05 levels except for the path from age to DB_W2. Indicators of all latent variables, correlations among the factors of family functioning, and correlations among covariates were omitted for clarity. DB_W2, delinquency at Wave 2; MU, mutuality; COM, communication; CONF, conflict and harmony; PCONC, parental concern; PCONT, parental control; FF, family functioning; BO, bonding; RES, resilience; SC, social competence; RPB, recognition for positive behavior; EC, emotional competence; CC, cognitive competence; BC, behavioral competence; MC, moral competence; SD, self-determination; SE, self-efficacy; CPI, clear and positive identity; BF, beliefs in the future; PI, prosocial involvement; PN, prosocial norms; SP, spirituality; PYD, positive youth development; Age, age; Sex, sex; and DB_Wl, delinquency at Wave 1.

To further understand the effect of Wave 1 family functioning on the *change* in Wave 2 delinquency, we controlled for Wave 1 delinquency as well (Figure 3). The model had a good fit to the data (χ^2^ = 83,575.5, *df* = 9,416, *p* < 0.001, NNFI = 0.98, CFI = 0.98, RMSEA = 0.049, SRMR = 0.056). The fit of this model was similar to the mediation model without covariates. The inclusion of covariates did not make a great change in the predictive paths among family functioning, PYD, and delinquency at Wave 2. According to Table 2, the indirect effect of family functioning at Wave 1 on delinquency at Wave 2 via PYD at Wave 2 was negative and statistically significant (β = −0.055, 95% CI = −0.059 to −0.051). The path coefficient of the direct effect of family functioning at Wave 1 on delinquency at Wave 2 decreased when controlling for PYD at Wave 2 (β = −0.031, *p* < 0.05) compared to the direct effect without controlling for PYD (β = −0.086, *p* < 0.05), indicating that effect of family functioning at Wave 1 on delinquency at Wave 2 is *partially mediated* by PYD at Wave 2 (66). The indirect effect explained 64.0% of the total effect. Overall, the mediation model explained 16.7% of the variance in delinquency at Wave 2, which was higher than the proportion of variance in delinquency at Wave 2 that was explained by the direct effect model without including PYD (10.0%). In sum, the mediation model held all relationships of interest when potential covariates were added.

## Discussion

In view of the paucity of research on the interrelationships amongst family functioning, PYD attributes, and adolescent delinquency (14, 29), the present study examined this question with particular reference to the mediating role of PYD attributes in the link between family functioning and adolescent delinquency. As previous studies on the relationships amongst family functioning, PYD, and adolescent delinquency covered only one or few aspects of family functioning and PYD (34, 67), we included different dimensions of family functioning and PYD in this study, which can be regarded as a methodological advance. Besides, as there are few longitudinal studies in this area in China, we adopted a short-term longitudinal design in this study, with a large sample recruited from Sichuan, China.

Regarding the question on the relationship between family functioning and adolescent delinquency (Research Question 1), our findings showed that family functioning negatively predicted adolescent delinquency over time after controlling for age, gender, and early delinquency scores, hence supporting Hypothesis 1. This finding echoes the thesis that family plays a protective role in reducing delinquent acts of adolescents (15). Also, the finding is consistent with the existing models on family functioning [e.g., (18–20)]. Moreover, it is in line with the previous studies in Western and non-Western contexts [e.g., (68, 69)] that family functioning is inversely related to adolescent delinquency. Zhu and Shek (29) further showed that there was parallel development of parental behavioral control (i.e., parenting measure) and adolescent delinquency. As most of the previous studies conceptualized family functioning in terms of a dimension, such as attachment, communication within the family (67) or parenting style (69), we operationalized family functioning in terms of an aggregation of different dimensions of family interaction and the parenting style. This approach is consistent with the view that family functioning is a multifaceted concept including interaction patterns and parenting style (70).

For the second research question, we found support for Hypothesis 2 that family functioning is a predictive factor of PYD attributes. This finding is in line with Hobfoll’s (71) Conservation of Resources theory which posits that personal and social resources tend to generate gains in other resources and these additional resources subsequently foster adaptation to adversity and well-being of people. In this study, interpersonal resources such as support from family and positive family interaction facilitated the development of personal resources like positive attributes of adolescents. Also, it echoes the findings of Yu et al. (45) which revealed the positive contribution of family cohesion and adaptability to psychological capital like self-efficacy and resilience of 1,971 Chinese college students. As pointed out by Shek et al. (44), while there are many studies investigating the impact of PYD attributes on adolescent developmental outcomes, there are relatively few studies examining the antecedents of PYD attributes, particularly family factors. As such, the present study broadens our understanding that family functioning plays an important role in the development of PYD attributes.

Regarding the relationship between PYD attributes and adolescent delinquency (Research Question 3), we found a significant negative relationship which provided support for Hypothesis 3. This finding is consistent with Reckless’s (7) containment theory of juvenile delinquency that the inner strength of the child is crucial to buffer against the pull toward delinquent engagement of the child. The present findings also provide support for the basic thesis of the PYD approach that developmental assets can protect adolescents from engaging in problem behavior (30). It also replicates the findings of Zhu and Shek’s (14) pioneer study that the PYD inversely predicted delinquency acts in Chinese adolescents over time.

Finally, the present findings supported for the partial mediating role of PYD attributes in the relationship between family functioning and adolescent delinquency over time. It supported Hypothesis 4. This observation is consistent with Mwangangi’s (15) model regarding the role of family functioning on adolescent delinquency which posits that a healthy family tends to promote the character, value, and skill development of the child, which in turn results in less delinquent acts of the child over time. The mediating role of PYD attributes also corroborates previous findings (43). As few studies have examined the mediating role of PYD attributes, this is an important addition to the literature (14, 29).

In previous studies, gender was found to be associated with PYD attributes and delinquency of Chinese adolescents [e.g., (14, 53)]. To advance our understanding on the interrelationships amongst family functioning, PYD attributes, and adolescent delinquency, gender difference in the mediation model of this study was explored. The results of the test for the gender invariance in the mediation model using multigroup analyses revealed that the mediating paths were invariant across gender [Δχ^2^ (2) = 7,250.4, *p* < 0.001, ΔCFI = 0.002, ΔRMSEA = 0.001]. As such, the mediating effect of PYD attributes in family functioning-adolescent delinquency link exists in both sexes and we can use the whole sample to examine the research questions.

Theoretically, this study highlights the importance of family functioning as a protective factor in adolescent delinquency. According to Sogar (16), most prominent theories in explaining the impact of family functioning on adolescent delinquency have mainly concerned with the impact of family adversity on adolescents, such as disrupted family relational processes and a lack of attachment in the social control theory (72), absence of a parent in the parental absence theory and family poverty in the economic strain theory (73). Unfortunately, the protective role of positive family functioning in the development of delinquent acts of adolescents has relatively received scant research attention. The present study illustrates how healthy family environment helps to prevent adolescents from engaging in delinquent acts via the development of adolescents’ positive attributes. The findings provide empirical support for Mwangangi’s (15) model of family functioning’s role in reducing adolescent delinquency where the family fosters the character, value, and skill development of the child which eventually contributes to a reduction in delinquent acts later. The findings also provide support for the family functioning models and the family ecological perspectives in the literature. Based on the ecological perspective (4) and general systems approach (5), the development of adolescents is likely to be affected by other contexts like schools and communities in addition to families. Future research should examine how other contextual factors (e.g., school, communities) protect adolescents from engaging delinquency via the promotion of positive youth attributes. Practically, the findings suggest that adolescent delinquency could be reduced by specialized intervention programs in family functioning and PYD. Some previous research on delinquent youth have illustrated that family-based interventions like functional family therapy would reduce subsequent delinquent acts of adolescents (74–76). Besides, van der Pol et al. (77) compared the effectiveness of multidimensional family therapy [targeted at the adolescent and his/her parent(s) and family] to individual psychotherapy like cognitive-behavioral therapy (exclusively focused on the adolescent) in the reduction of delinquent acts of 169 adolescents with cannabis use disorder. They reported that multidimensional family therapy was more effective than individual psychotherapy for violent crimes. This observation might be attributed to the fact that multidimensional family therapy has a more comprehensive focus and targets a larger number of empirically supported risk factors (i.e., attempting to change the parents, the youths, and their interactions in relation to the family and relative to the extrafamilial) than does a treatment focused primarily on the individual adolescent. However, as nearly half of the effect of family functioning on delinquency was attenuated when we control for personal PYD attributes, this finding suggests that both personal and contextual factors are important to prevent adolescents from engaging in delinquency. This conceptualization is in contrast to some theories of crime (e.g., self-control theory of crime) which view the individual propensity as the sole factor to account for delinquency [e.g., (10)]. Interventions targeting at fostering both healthy family environments (e.g., Functional family therapy and Parent-child interaction therapy) and positive attributes of adolescents (e.g., leadership training, resilience programs) are recommended to help reduce adolescent delinquency in China. Serving as the important socializing agent for the development of adolescents, schools may also regularly provide seminars to parents and enhance their parenting styles and communicative skills. At the same time, life and moral education in schools may serve as important agent to assist adolescents in fostering positive youth attributes. In fact, research showed that different stakeholders regard PYD attributes (such psychosocial competence) as very important for adolescent thriving but the related education is inadequate in the school context (78).

In the light of the significant mediating role of PYD underlying the impact of family functioning on adolescent delinquency in later times, the development of positive attributes among adolescents could prevent adolescents from engaging in delinquent acts in later times. However, evidence-based holistic PYD programs for adolescents in mainland China are sparse (37). Based on the positive outcomes of the Project P.A.T.H.S. in Hong Kong, we developed and implemented the Tin Ka Ping P.A.T.H.S. Program in mainland China. Zhu and Shek (79) conducted a pioneer study to investigate the effectiveness of a PYD program (“Tin Ka Ping P.A.T.H.S. Project”) on life satisfaction, depression, and delinquency prior to and after project implementation of 1,044 adolescents in mainland China. They revealed a significant decrease in delinquency of the experimental group, but not in control counterparts, hence underscoring the importance of promotion of PYD attributes in adolescents as a strategy to deal with the issue of adolescent delinquency.

Despite the pioneer nature of this study in the Chinese context, there are several limitations in this study. First, the mediation model was developed based on two waves of data only. To delineate a more comprehensive picture of the causal influence of the mediator, future research should collect more waves of data in a longer time span. Second, as this study is confined to adolescents in Sichuan, China, generalizability of the findings to adolescents in other Chinese communities is not knowable at this stage. Future research should replicate the study in other Chinese contexts. This would help support the external validity and reinforce the credibility of the conclusions of this study. Third, as self-reported measures were utilized to collect data at Wave 1 and Wave 2, there is a risk of common method variance bias which poses the threat to the validity of the present findings (80). The correlations between variables might be artificially inflated or deflated because of the common method used to collect data. Therefore, future research should collect data through multiple informants (e.g., parents, teachers and peers) as well as different measuring methodologies (e.g., objective observation).

In conclusion, as stated by Ye (81), there is an increase in juvenile offenders in China since 2018. In 2019, the country saw 61,295 minors reviewed for prosecution, which is 5.12% higher than the year before. As such, prevention of adolescent delinquency is of paramount importance. The present study addressed conceptual and methodological limitations in the scientific literature on the prevention of delinquency of Chinese adolescents. Conceptually, we adopted a positive view (rather than a deficits view) of adolescent delinquency and PYD approach in this study. Methodologically, we recruited a large sample of Chinese adolescents and utilized validated measures via a longitudinal research design. Consistent with our expectations, this study revealed that family functioning helps reduce adolescent delinquency via the promotion of positive youth attributes of adolescents. As such, both family environments and positive attributes of adolescents are important to consider for any intervention measure to adolescent delinquency.

## Data Availability Statement

The raw data supporting the conclusions of this article will be made available by the authors, without undue reservation.

## Ethics Statement

The studies involving human participants were reviewed and approved by Institutional Review Board of Sichuan University. Written informed consent to participate in this study was provided by the participants’ legal guardian/next of kin.

## Author Contributions

DS, XZ, and DD: conceptualization, methodology, and writing—review and editing. KL and DS: formal analysis and writing—original draft preparation. All authors have read and agreed to the publication of this manuscript.

## Conflict of Interest

The authors declare that the research was conducted in the absence of any commercial or financial relationships that could be construed as a potential conflict of interest.

## Publisher’s Note

All claims expressed in this article are solely those of the authors and do not necessarily represent those of their affiliated organizations, or those of the publisher, the editors and the reviewers. Any product that may be evaluated in this article, or claim that may be made by its manufacturer, is not guaranteed or endorsed by the publisher.
